# The Pathogenicity of Anti-*β*2GP1-IgG Autoantibodies Depends on Fc Glycosylation

**DOI:** 10.1155/2015/638129

**Published:** 2015-06-22

**Authors:** Christoph Fickentscher, Iryna Magorivska, Christina Janko, Mona Biermann, Rostyslav Bilyy, Cecilia Nalli, Angela Tincani, Veronica Medeghini, Antonella Meini, Falk Nimmerjahn, Georg Schett, Luis E. Muñoz, Laura Andreoli, Martin Herrmann

**Affiliations:** ^1^Department for Internal Medicine 3, University Hospital Erlangen, Friedrich-Alexander-Universität Erlangen-Nürnberg (FAU), Ulmenweg 18, 91054 Erlangen, Germany; ^2^Institute of Cell Biology, National Academy of Sciences of Ukraine, Drahomanov Street 14/16, Lviv 79005, Ukraine; ^3^Department of Otorhinolaryngology, Head and Neck Surgery, Section for Experimental Oncology and Nanomedicine (SEON), University Hospital, Waldstraße 1/Glückstraße 10, 91054 Erlangen, Germany; ^4^Rheumatology and Clinical Immunology, Department of Clinical and Experimental Sciences, Spedali Civili and University of Brescia, Piazzale Spedali Civili 1, 25123 Brescia, Italy; ^5^Paediatric Immunology and Rheumatology, Children's Hospital Brescia, Department of Clinical and Experimental Sciences, Spedali Civili and University of Brescia, Piazzale Spedali Civili 1, 25123 Brescia, Italy; ^6^Department of Biology, Institute of Genetics, Friedrich-Alexander-Universität Erlangen-Nürnberg (FAU), Erwin-Rommel Str. 3, 91058 Erlangen, Germany

## Abstract

To analyze the glycosylation of anti-*β*2GP1, we investigated purified IgG from healthy children, patients with APS, and asymptomatic adult carriers of antiphospholipid antibodies. We observed that in the sera of healthy children and of patients with APS, IgG3 and IgG2 were predominant, respectively. The potentially protective anti-*β*2GP1-IgM was lower in the sera of healthy children. Although anti-*β*2GP1-associated C1q did not differ between children and patients with antiphospholipid syndrome, the associated C3c was significantly higher in the sera of healthy children. This indicates a more efficient clearance of anti-*β*2GP1 immune complexes in the healthy children. This clearance is not accompanied by inflammation or coagulatory events. It is likely that the most important pathogenic factor of the anti-*β*2GP1-IgG is related to the different glycosylation observed in healthy and diseased individuals. We detected a significantly higher sialylation of anti-*β*2GP1-IgG isolated from the sera of healthy children and asymptomatic adults when compared with that of patients with clinically apparent antiphospholipid syndrome. Low sialylated IgG reportedly ameliorates inflammation and inflammation promotes hyposialylation. Thus, both reactions create a vicious circle that precipitates the pathology of the antiphospholipid syndrome including thrombus-formation. We conclude that the increased sialylation of anti-*β*2GP1-IgG of sera of healthy individuals limits their pathogenicity.

## 1. Introduction

The antiphospholipid syndrome (APS) is an autoimmune disease affecting the coagulation system. Its diagnosis requires at least one clinical criterion and one serological criterion as defined during conferences held in 1998 and in 2006 [1, 2]. The serological criteria include the detection of so-called antiphospholipid antibodies (aPL) which comprise the lupus anticoagulant (LA), anticardiolipin autoantibodies (aCL), and anti-*β*2-glycoprotein 1 autoantibodies (anti-*β*2GP1) [1].

Thrombosis as one major symptom in APS is mainly associated with anti-*β*2GP1 [3]. *β*2GP1 is a plasma protein composed of five domains [4] and it is known to be an inhibitor of the contact activation of the intrinsic coagulation pathway [5]. Additionally, aPL can cause a prothrombotic state in individuals by activating platelets and endothelial cells resulting in a higher expression of tissue factor or in the upregulation of proinflammatory cytokines and cell adhesion molecules [6, 7].

Patients with APS can be classified into two groups depending on the occurrence of further diseases: (I) patients merely suffering from APS (primary antiphospholipid syndrome; PAPS) and (II) those with an underlying systemic lupus erythematodes (SLE). The latter condition is referred to as secondary antiphospholipid syndrome (SAPS) [8].

Up to now, a variety of hypotheses about possible triggers for the induction of anti-*β*2GP1 have been proposed. Published studies suggest that their production can be provoked during infectious diseases like parvovirus B19, HIV, CMV, or HCV [3, 9–11]. Certain molecular structures of these infectious agents may resemble structures of *β*2GP1 and thus cause the induction of cross-reacting antibodies [7, 12–16]. Another one claims that nutritional exposure to *β*2GP1 could also trigger the production of transient and harmless anti-*β*2GP1 in children [4, 17–19].

Interestingly, anti-*β*2GP1 can be detected in a quite large proportion of the healthy population who do neither suffer from APS nor another autoimmune disease [3, 18, 20]. As complication-free periods of patients with APS occur although the aPL are persistently detectable in their circulation [15], it seems to be likely that the mere presence of these antibodies is not sufficient for the development of clinical active APS complications.

For this reason, a two-hit hypothesis has been proposed to provide a putative explanation for this observation. (I) the production of anti-*β*2GP1 represents an initial hit that increases the risk for thrombotic events and (II) infectious agents can then serve as the second hit provoking the typical manifestations of APS by activating toll-like receptors or complement [9, 15, 21, 22]. This two-hit hypothesis surely provides a good first model for the etiopathogenesis of APS. Yet it is not completely satisfying and does not explain why some people harbouring aPL remain healthy. Some study results indicate that this could be grounded in structural differences of anti-*β*2GP1 found in patients with APS and healthy children [4, 23].

Comparing obviously harmless aPL from healthy children with putatively pathogenic aPL from adults with APS, we suggested a difference in the epitope specificity as differentiating factor. Anti-*β*2GP1 from the sera of the children specifically bound to the domain IV/V of *β*2GP1, whereas anti-*β*2GP1 from adults with APS preferentially targeted domain I [24]. These results were consistent with those described in other studies [19, 25]. Besides distinct epitope binding specificities, a different composition of the spectrum of the anti-*β*2GP1 IgG subclasses between patients with APS and healthy carriers of this antibody has also been described [19, 23, 26, 27].

The differences in glycosylation of antibodies which are accompanied by crucial modifications of their effector functions [28] provide another promising approach in performing structural analyses of anti-*β*2GP1. IgG molecules possess a biantennary oligosaccharide attached to the asparagine at position 297 of the Fc portion (Asn-297). Various glycoforms containing distinct sugar moieties have been described [28, 29]. Interestingly, it has been observed that IgG molecules whose glycans terminate with sialic acid residues show a decreased affinity to activating Fc*γ*-receptors (Fc*γ*Rs) while simultaneously provoking an upregulation of the inhibitory Fc*γ*RIIB [29–31]. These effects lead to a rather anti-inflammatory activity and might consequently be able to prevent potentially harmful immune responses.

In particular in children, aPL can readily be detected although they do not show any clinical signs of APS. In a study carried out by Avčin et al., the prevalence of anti-*β*2GP1 in 61 healthy children was 6.6% [18]. Based on this observation we intended to find out why these circulating autoantibodies do not cause harm in their hosts. We determined the IgG-subclass distribution in the samples from healthy children and patients with APS and examined the binding of the complement factors C1q and C3c to anti-*β*2GP1 immune complexes, since an important involvement of the complement system in the pathogenesis of APS has been reported [21, 22, 32]. Finally we analysed the sialylation status of anti-*β*2GP1-IgG isolated from healthy children, asymptomatic adult carriers, and patients with APS.

Our results revealed that, compared to adults with APS, the healthy children showed a higher content of IgG3 at the cost of IgG2 of the anti-*β*2GP1. Furthermore, anti-*β*2GP1-IgG of healthy children showed a higher sialylation and displayed more bound C3c. Hyposialylated IgG and inflammation mutually influence the pathogenicity of the antiphospholipid autoantibodies and form a vicious pathogenic circle.

## 2. Materials and Methods

### 2.1. Patients

For the investigation of anti-*β*2GP1-IgG, its subclasses, C1q- and C3c-binding and anti-*β*2GP1-IgM, we studied sera of 46 adult patients (24 patients with PAPS and 22 with SAPS) and 16 healthy children harbouring anti-*β*2GP1 without any clinical manifestations associated with APS from our partner hospital in Brescia, Italy. As a control group, we examined sera of 17 age and sex matched adult normal healthy donors (NHD) without aPL. Inclusion criteria for the children were the presence of anti-*β*2GP1-IgG, no infectious diseases in the course of three months before blood taking, and no chronic pathology. Additionally, it was excluded that their parents suffered from any autoimmune disease. At the time of blood taking, the mean age of the children was about 9 months (median age: 8.5 months; range: 3 months and 5 days–19 months and 10 days).

Since sugar residues attached to molecules can be damaged by repeated thawing and freezing, we received new sera from our partner hospital in Brescia, Italy, to analyse the sialylation of anti-*β*2GP1-IgG. We obtained sera from 12 patients with PAPS and 10 patients with SAPS, 15 patients with SLE and aPL but without clinical evidence of APS and sera from 6 asymptomatic carriers of aPL. Furthermore, we obtained sera from 16 healthy children showing no symptoms of APS although harbouring anti-*β*2GP1-IgG in their circulation (mean age: 9.5 months; median age: 10 months; range: 3 months and 5 days–19 months and 10 days).

### 2.2. Blood Drawing

Heparinised venous blood was drawn by venipuncture and processed within two hours. Serum was obtained by centrifugation at 1000 g for 15 minutes of clotted blood, then aliquoted, frozen, and stored at −20°C until use for investigation.

Informed consent was obtained from all blood donors and the children's parents, respectively, and the study was approved by the ethics committees of the University Hospital Erlangen and the Spedali Civili Brescia.

### 2.3. Isolation of *β*2GP1

For *β*2GP1-isolation from diluted sera of normal healthy donors (in 0.15 M NaCl), percloric acid was slowly added until the mixture got a creamy aspect. After centrifugation at 10.000 g, the supernatant was neutralized with NaOH and dialyzed against a buffer of 0.02 M Tris and 0.03 M NaCl, pH 8. Then it was given into columns containing HiTrap heparin and contaminants of the supernatant were eluted by the addition of 0.02 M Tris and 0.15 M NaCl, pH 8. In order to elute semipurified *β*2GP1, a buffer of 0.02 M Tris, and 0.35 M NaCl, pH 8 was injected into the columns and the fractions have been pooled and dialyzed against a solution containing 0.05 M acetate buffer, 0.05 M NaCl, pH 4.8. This material was filtrated with 0.2 *μ* membranes and injected into resin columns equilibrated with 0.05 M acetate buffer, 0.05 M NaCl, pH 4.8. After washing with 0.05 M acetate buffer, 0.65 M NaCl, pH 5.2, the fractions were pooled and dialyzed against PBS, pH 7.2. To verify the purity of isolated *β*2GP1, a sodium dodecyl sulfate polyacrylamide gel electrophoresis (SDS-PAGE) has been performed.

### 2.4. Purification of Anti-*β*2GP1-IgG

For the analysis of the sialylation of anti-*β*2GP1-IgG, we isolated IgG antibodies from the sera to exclude possible interactions of the detection lectin with anti-*β*2GP1-IgM in the samples. Purification of anti-*β*2GP1-IgG was performed as follows: 20 *μ*L of serum was added to 50 *μ*L magnetic Dynabeads Protein G (Novex by life technologies, Carlsbad, CA, USA) and incubated for 45 minutes at room temperature. After washing with 1x TBS, glycin-HCl, pH 2.6, was added for 5 minutes to elute the anti-*β*2GP1-IgG. The supernatant, now containing the IgG antibodies, was neutralized with Tris-HCl, pH 8.8, and all samples were centrifuged using 100 K Amicon Ultra-centrifugal filter devices (Millipore, Tullagreen, Ireland) to transfer them into 300 *μ*L TBS-buffer.

### 2.5. Serum ELISAs: Anti-*β*2GP1-IgG, -IgG1, -IgG2, -IgG3, -IgG4, and -IgM and C3c and C1q Bound to Anti-*β*2GP1

To quantify the amount of anti-*β*2GP1-IgG and its four subclasses, of C3c and C1q associated with anti-*β*2GP1 and of anti-*β*2GP1-IgM in the sera of patients with PAPS and SAPS, NHD, and healthy children, we performed enzyme-linked immunosorbent assays (ELISA). In detail, the surface of F 96 NUNC maxisorp microtiter plates (Thermo Scientific, Roskilde, Denmark) was coated with 50 *μ*L of a 2 *μ*g/mL solution of *β*2GP1 in coating buffer (100 mM Na_2_CO_3_, 100 mM NaHCO_3_, pH 9.6) at 4°C overnight. After washing the plates three times with washing buffer (WB; 0.05% Tween 20, 0.1 mM CaCl_2_, 0.1 mM MgCl_2_ in TBS buffer (150 mM NaCl, 50 mM TrisHCl, pH = 7.4)), the wells of the coated plates were blocked with 100 *μ*L/well of a 2% BSA solution for two hours at 37°C. After three further washing steps with WB, 50 *μ*L/well of diluted serum (1 : 200 in WB) was added and the plates were incubated at 37°C for two further hours. Then, the plates were washed three times with WB again.

Development of the plates based on an enzymatic reaction of horseradish peroxidase (HRP) with TMB as chromogenic substrate. Therefore, the appropriate HRP-conjugated detection-antibody was added as a next step (Table 1). After incubation at 37°C for another hour and three washing steps with WB, the plates were developed by the addition of 50 *μ*L/well of freshly prepared TMB-solution (1 mL of 0.01 g 3,3′,5,5′-tetramethylbenzidine (Sigma-Aldrich, Steinheim, Germany) dissolved in 10 mL dimethyl sulfoxide, mixed with 9 mL of 0.1 M Na_2_HPO_4_, 0.05 M citric acid, pH 4.5–5.5, and 2 *μ*L of H_2_O_2_). The reaction was stopped by adding 50 *μ*L/well of 25% sulfuric acid (H_2_SO_4_). Finally, optical density (OD) was measured at 450 nm and 650 nm as correction factor using an infinite F200 pro ELISA-reader (Tecan).

After subtraction of the corresponding blank values of the plates, experimental values were expressed as optical density or as mean optical density of each serum when ELISA tests were performed in monoplicates or duplicates, respectively.

### 2.6. IgG-ELISA: SNA

In order to examine the sialylation of anti-*β*2GP1-IgG, we slightly modified the previously described ELISA protocol. Before coating, the *β*2GP1 antigen was treated with 1% of periodic acid (PIA, Sigma-Aldrich, Steinheim, Germany) for 48 hours at 43°C to remove its sugar residues to exclude unspecific binding of* Sambucus nigra* lectin (SNA) to naive *β*2GP1. After that, we centrifuged the *β*2GP1-solution with 10 K Amicon Ultra-centrifugal filter devices (Millipore, Tullagreen, Ireland) to transfer the treated *β*2GP1 into coating buffer (100 mM Na_2_CO_3_, 100 mM NaHCO_3_, pH 9.6).

Then, the plates were coated with 50 *μ*L/well of a 2 *μ*g/mL solution of the PIA-treated *β*2GP1 at 4°C overnight and subsequently blocked for two hours at 37°C with 100 *μ*L/well of PIA-treated (0.5%) 3% gelatine (Sigma-Aldrich, Steinheim, Germany) in TBST buffer with 1 mM CaCl_2,_ and 1 mM MgCl_2_. The subsequent steps were performed as described in the previous ELISA protocol with two exceptions. Instead of serum, we added 50 *μ*L/well of purified IgG-samples to exclude possible binding of SNA to anti-*β*2GP1-IgM. And the development of the plate required the addition of a second detection reagent (Table 2), which was incubated for one hour at room temperature and demanded three preliminary washing steps. We calculated the SNA/anti-*β*2GP1-IgG ratio from the OD values we obtained in the SNA ELISA and a simultaneously conducted anti-*β*2GP1-IgG ELISA.

### 2.7. Statistical Analysis

Differences between the cohorts were assessed using the one-way analysis of variance (ANOVA) test. In the case of multiple testing, the significance values were adjusted by the Newman-Keuls posttest correction.

## 3. Results

### 3.1. Analysis of Anti-*β*2GP1-IgG

**Figure 1:**
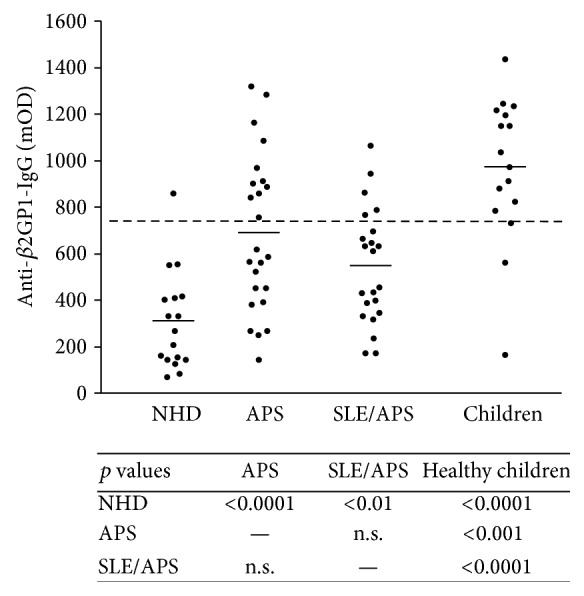
Total anti-*β*2GP1 IgG in the sera of NHD, PAPS, SLE/APS, and healthy children and the corresponding *p* values. NHD showed significantly lower mOD values than all other cohorts whereas healthy children represented the highest mOD values. Short horizontal lines: mean mOD values of all samples of the cohort. Long broken line: 97.5% quantile of NHD (=738 mOD); NHD = normal healthy donor; PAPS = primary antiphospholipid syndrome; SLE/APS = antiphospholipid syndrome with systemic lupus erythematosus; OD = optical density.

We measured the OD values of total anti-*β*2GP1-IgG in the sera of 24 patients with PAPS and 22 patients with SLE/APS, 16 healthy children, and 17 NHD as controls. As shown in Figure 1, the lowest IgG values were to be observed in the cohort of healthy donors (308 mOD), whereas the values of the healthy children (970 mOD) even exceeded that of the patients with APS (PAPS: 686 mOD, *p* = 0.010; SLE/APS: 546 mOD, *p* < 0.001). All differences between NHD and the other cohorts as well as between the children and the other cohorts were significant. Patients with PAPS and SLE/APS did not differ significantly.

We calculated the 97.5% quantile of the anti-*β*2GP1-IgG OD values obtained from the NHD cohort and set this value as cut-off (738 mOD). Sera exceeding this value were considered seropositive for anti-*β*2GP1-IgG. Applying this cut-off value, we obtained 31 seropositive samples (12/24 PAPS, 6/22 SAPS, 13/16 healthy children), which were further analysed in our study (Supplementary Figure 1, in Supplementary Material available online at http://dx.doi.org/10.1155/2015/638129).

### 3.2. Analysis of the IgG-Subclasses of Anti-*β*2GP1

**Figure 2:**
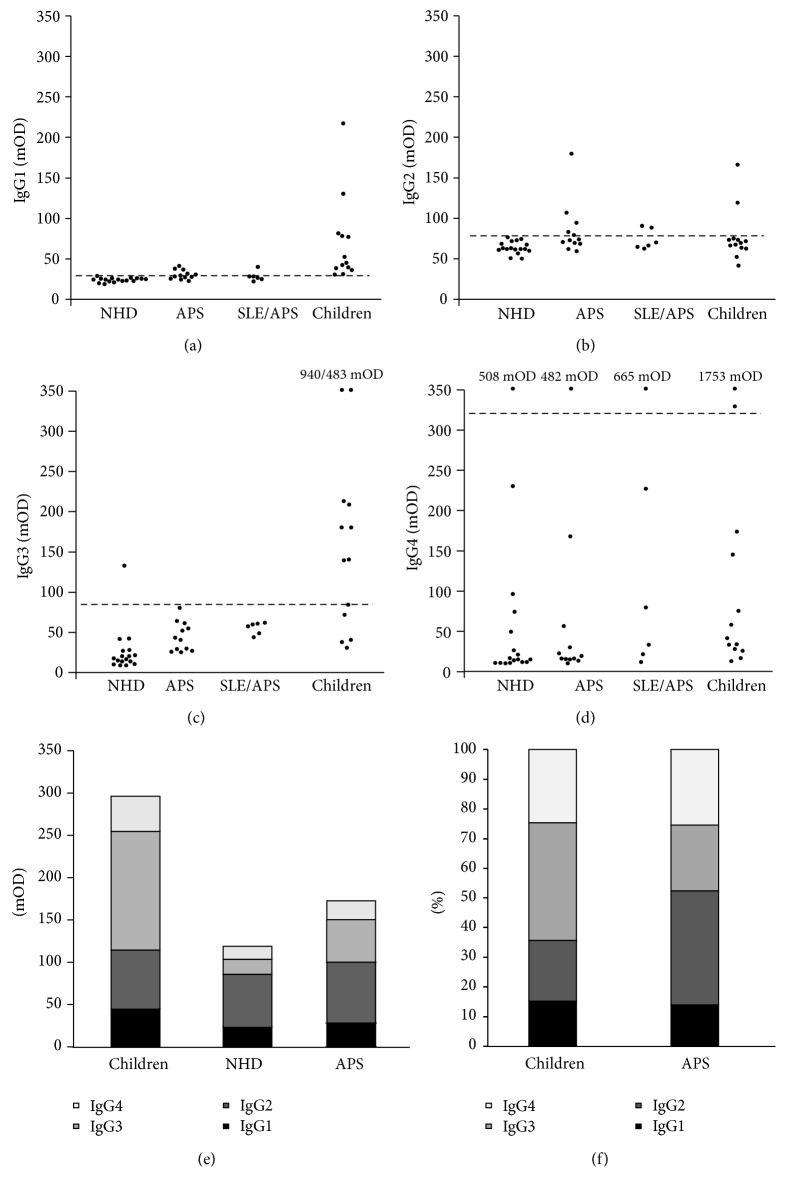
mOD values of anti-*β*2GP1-IgG1 (a), anti-*β*2GP1-IgG2 (b), anti-*β*2GP1-IgG3 (c), and anti-*β*2GP1-IgG4 (d) in NHD, PAPS, SLE/APS, and children. The horizontal lines represent the cut-off for seropositivity of the corresponding anti-*β*2GP IgG-subclass (>mean mOD_[NHD]_ + 2 ×  SD_[NHD]_); NHD = normal healthy donor; PAPS = primary antiphospholipid syndrome; SLE/APS = antiphospholipid syndrome with systemic lupus erythematosus; OD = optical density. Median OD values of the anti-*β*2GP1-IgG subclasses in the study cohorts (e). Regarding the anti-*β*2GP1-IgG subclass-distribution within the cohorts, children showed a significantly higher contribution of IgG3 than IgG1 and IgG2. Patients with antiphospholipid syndrome (APS) showed a higher content of anti-*β*2GP1-IgG2 than -IgG1 and -IgG3. NHD showed a higher contribution of anti-*β*2GP1-IgG2 than -IgG1, -IgG3, and -IgG4. The columns represent the median OD values of IgG1, IgG2, IgG3, and IgG4 in the cohorts. OD = optical density; NHD = normal healthy donor. Percentage distribution of anti-*β*2GP1-IgG subclasses in children and patients with APS (f). Children harbour significantly less anti-*β*2GP1-IgG2 than patients with APS but more anti-*β*2GP1-IgG3. APS = antiphospholipid syndrome.

Analysing the absolute OD values of the anti-*β*2GP1-IgG subclasses, we observed that the children showed significantly higher levels of the anti-*β*2GP1-IgG1 (69 mOD) and -IgG3 subclasses (212 mOD) compared to NHD, PAPS, and SAPS (Figures 2(a) and 2(c); Table 3(a)). The differences between healthy children and NHD regarding these two subclasses showed a higher level of significance than the comparison between healthy children and patients with APS. However, healthy children did not show significant differences for anti-*β*2GP1-IgG2 and -IgG4 when compared to NHD, PAPS, and SAPS (Figures 2(b) and 2(d); Table 3(b)).

Patients with APS (PAPS and SAPS) showed significantly higher OD values for anti-*β*2GP1-IgG1, -IgG2, and -IgG3 than NHD (Figures 2(a), 2(b), and 2(c); Table 3(b)). As in the anti-*β*2GP1-IgG ELISA, we observed no significant difference between the patients with PAPS and SAPS.

To summarize, patients with APS showed higher OD values than NHD in IgG1, IgG2, and IgG3. IgG4 did not show any significant difference. The children, in turn, had the highest OD values of the IgG1 and IgG3 subclasses.

These results were consistent with the analysis of the number of seropositive samples, as shown in Table 4. To determine seropositivity, we calculated the mean OD and the standard deviation (SD) of the NHD group for the different IgG subclasses. IgG values exceeding the NHD's mean OD plus two times the SD of the considered subclass were defined as seropositive (Supplementary Figure 1). Applying this cut-off, we observed that the healthy children had high levels of the IgG1 and IgG3 subclasses, with 13 (100%) and 8 (62%) seropositive samples, respectively. In contrast, only 7 (39%) and 0 (0%) sera of patients with APS were seropositive for IgG1 and IgG3, respectively. APS showed seropositivity of anti-*β*2GP1-IgG2 in 7 cases (39%), whereas the cohort of healthy children contained just 2 seropositive sera (15%). Just few samples were seropositive for anti-*β*2GP1-IgG4 in each cohort, representing a range from 8% (PAPS) to 17% (SAPS).

To determine the subclass distribution within the cohorts, we calculated the contribution of the mean OD values of each individual subclass to the sum.

As shown in Figures 2(e) and 2(f), we saw that for the healthy children IgG3 was the most abundant subclass with a contribution of 40% (IgG3 > IgG1 (*p* < 0.001) and IgG3 > IgG2 (*p* < 0.01)). In contrast, IgG2 dominated the subclass profile for NHD (46%; IgG2 > IgG1, IgG2 > IgG3, and IgG2 > IgG4 (*p* < 0.0001)) and patients with APS (38%; IgG2 > IgG1 (*p* < 0.0001), IgG2 > IgG3 (*p* = 0.001)). The contribution of IgG1 ranged from 12% (SAPS) to 17% (NHD) and that of IgG4 from 21% (PAPS) to 35% (SAPS). Comparing the contribution of the individual subclasses between the study cohorts, we observed that children showed significantly less IgG2 than patients with APS and NHD (*p* < 0.001; *p* < 0.0001) and a higher IgG3 content than NHD and patients with APS (*p* < 0.0001; *p* < 0.001). No significant difference between the cohorts was to be seen for the contributions of IgG1 and IgG4.

### 3.3. Analysis of IgM, C1q, and C3c Associated to Anti-*β*2GP1-IgG

Next we analysed potential *β*2GP1-binding proteins in the sera of healthy children and adults with APS. The signals for C1q were low and did not differ significantly between the cohorts.

**Figure 3:**
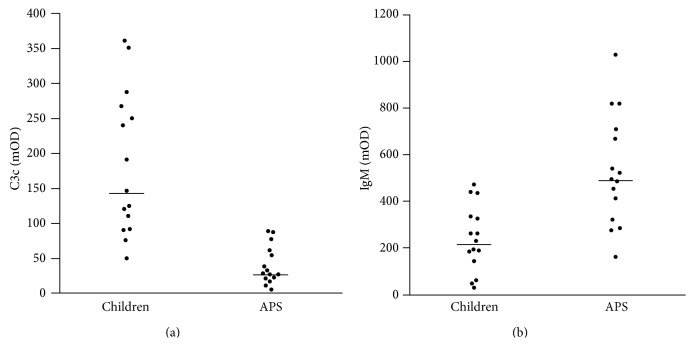
OD values of C3c (a) bound to anti-*β*2GP1 and OD values of anti-*β*2GP1-IgM (b) in sera of patients with APS and healthy children. Anti-*β*2GP1 of healthy children displayed significantly more bound C3c than that of patients with APS (*p* = 0.00007). This suggests a more efficient clearance of *β*2GP1-immune complexes in the healthy children. Anti-*β*2GP1-IgM OD values of patients with APS were significantly higher than those of healthy children. Horizontal lines represent the median OD value of the cohort. APS = antiphospholipid syndrome; OD = optical density.

However, the ELISAs detecting C3c bound to anti-*β*2GP1 and anti-*β*2GP1-IgM showed highly significant differences between healthy children and adults with APS. Whereas C3c-anti-*β*2GP1 adducts were increased in healthy children (*p* < 0.0001; Figure 3(a)), anti-*β*2GP1-IgM was decreased (*p* < 0.001; Figure 3(b)).

### 3.4. Analysis of the Sialylation of Anti-*β*2GP1

We analysed the glycosylation of anti-*β*2GP1-IgG, focusing on its sialylation since highly sialylated Fc-parts of IgG are known to cause rather anti-inflammatory responses [30]. We measured the binding of the sialic acid specific lectin SNA to the anti-*β*2GP1-IgG. As shown in Table 5, we employed five cohorts of individuals: 12 patients with PAPS, 10 patients with SAPS (SLE as primary disease), 15 patients with SLE who did not show clinical sign of APS but with aPL in their sera (SLE + aPL), 6 asymptomatic adult carriers of aPL (aaPL), and 16 healthy children, harbouring aPL.

**Figure 4:**
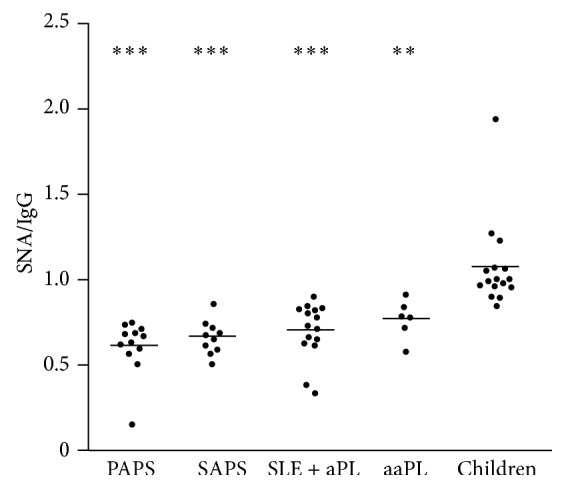
SNA/anti-*β*2GP1-IgG ratio in PAPS, SAPS, SLE + aPL, aaPL, and children. Healthy children show a significantly higher SNA/anti-*β*2GP1-IgG ratio than PAPS, SAPS, SLE + aPL, and aaPL (^*∗∗*^ *p* < 0.0001; ^*∗∗∗*^ *p* < 0.00001). This suggests higher sialylation of the oligosaccharides attached to Fc fragments of the children's anti-*β*2GP1. No significant differences were found between the remaining cohorts. Horizontal lines indicate the mean SNA/IgG ratio of the cohort. SNA =* Sambucus nigra* agglutinin; APS = antiphospholipid syndrome; PAPS = patients with primary APS; SAPS = patients with APS and SLE as underlying disease; SLE + aaPL = patients with SLE without symptoms of APS harbouring circulating aPL; aaPL = asymptomatic carriers of aPL; aPL = antiphospholipid antibodies; SLE = systemic lupus erythematosus.

Analysing the SNA/IgG-ratios (Figure 4), we identified the highest sialylation level in the sera of the healthy children. The significances were rather high and decreased from PAPS (*p* < 0.0001) to SAPS (*p* < 0.0001), to SLE + aPL (*p* < 0.0001), and to aaPL (*p* < 0.001).

## 4. Discussion

We analysed two distinct sets of sera. One was used to investigate anti-*β*2GP1-IgG, its subclasses, and its associated proteins C1q and C3c. For the analysis of the sialylation of anti-*β*2GP1-IgG, we had to collect fresh sera from patients with APS and the healthy children since the stability of the antibody-glycosylation is unknown. In order to avoid its possible decay by processes like enzymatic degradation, this period has been kept as short as possible and was followed by immediate investigation of the glycosylation.

The antiphospholipid syndrome is characterized by clinical manifestations such as arterial and venous thrombosis or pregnancy-related complications and the detection of specific autoantibodies (lupus anticoagulant, anti-*β*2GP1- and anticardiolipin-autoantibodies) [1]. In particular anti-*β*2GP1s are considered responsible for provoking the pathological complications [3]. We first measured the total amount of anti-*β*2GP1-IgG in the sera of 16 healthy children without clinical manifestations of APS and 46 adult patients with APS to identify individual cohorts seropositive for anti-*β*2GP1. 17 adult normal healthy donors served as negative control.

The samples of the patients with APS showed higher levels of anti-*β*2GP1-IgG than those of NHD. We also observed a higher concentration of anti-*β*2GP1-IgG in the sera of healthy children compared to NHD. This is due to the fact that the detection of these antibodies has been the inclusion criterion for the healthy children examined in this study. Interestingly, the children showed even higher OD values for the putatively pathogenic anti-*β*2GP1-IgG than the patients with APS. This finding was surprising since the children were clinically healthy and did not show any signs of APS such as thrombosis or thrombocytopenia, complications in which the existence of aPL, especially anti-*β*2GP1, is made responsible for [7, 33].

High levels of circulating aPL in 61 healthy children, when compared to healthy adults, have already been described by Avčin and colleagues [18]. The anti-*β*2GP1-IgG was reportedly higher in preschool children with a mean age of 5 years than in adolescents with a mean age of 13.5 years [18]. The strongly elevated circulating anti-*β*2GP1-IgG in our cohort of healthy children may be explained by the fact that they had an even lower age (mean age was nine months). One possible explanation for this result is the induction of anti-*β*2GP1 by viral and bacterial infections [10, 11] or nutritional exposure to *β*2GP1 [17]. As the immune system in children is still to evolve, they are more susceptible to infections than adults and their immune system has to cope with a plethora of viral and bacterial pathogens for the first time in their life. This scenario is prone to result in the stimulation of autoantibody-production. Molecular mimicry of certain pathogens can lead to the production of antibodies which then cross-react with *β*2GP1 [7, 12–15]. When infants diversify their dietary habits and start to consume products like milk and meat, they can get into contact with nutritional *β*2GP1 [19]. This is a further condition capable triggering the production of anti-*β*2GP1. Exposure to pathogen-derived and dietary *β*2GP1 could explain the high level of circulating anti-*β*2GP1-IgG in healthy children. This low affine reactivity is consecutively lost during the maturation of the immune system and replaced by the emergence in adults of high affine antibodies that more specifically recognize pathogens. Apart from possible origins of these autoantibodies, it is obvious that they do not provoke any of the hallmark symptoms of APS. In contrast to their adult counterparts, anti-*β*2GP1-IgGs circulating in the plasma of healthy children are obviously not pathogenic. So it seems decent to hypothesize that anti-*β*2GP1 autoantibodies in healthy children biochemically differ from those in adults.

To test this hypothesis, we examined the anti-*β*2GP1-IgG subclass distribution in the seropositive sera of the anti-*β*2GP1-IgG ELISA. The healthy children showed higher OD values for the IgG1- and IgG3-subclasses than patients with APS and NHD. This was consistent with the number of seropositive samples. In the anti-*β*2GP1-IgG1-subclass, every tested serum of the healthy children surpassed the threshold set for seropositivity (>mean OD_[NHD]_ + 2 ×  SD_[NHD]_) and 8 samples (62%) did so in the IgG3-subclass. In the sera of patients with APS, just 7 (39%) and no samples were seropositive for IgG1 or IgG3. In the sera of patients with APS compared to NHD, we observed higher OD levels of anti-*β*2GP1 in all subclasses, except for IgG4. This result is consistent with the findings of Guerin et al. who reported elevated levels in all anti-*β*2GP1-IgG subclasses, even including IgG4, in patients with APS compared to a control group of healthy adults [23].

It might be that the elevated absolute OD values of the anti-*β*2GP1-IgG subclasses are the logical consequence of the generally higher level of anti-*β*2GP1-IgG in the patients with APS and healthy children who were selected for further testing. Therefore, we also analysed the subclass distribution of this antibody. We first analysed the anti-*β*2GP1-IgG subclass distribution within the individual cohorts and observed that in the healthy children the IgG3 antibody was most abundant with a contribution of 40% to the total reactivity. This is consistent with the finding that all sera of the healthy children were seropositive in this subclass and that their anti-*β*2GP1-IgG3 OD values were the highest of all cohorts. This indicates a predominance of this subclass in the healthy children. The remaining anti-*β*2GP1-IgG subclasses in the sera of the children ranged from 15% (IgG1) over 20% (IgG2) to 25% (IgG4) but the differences between their proportions were not statistically significant. In contrast, in the sera of the patients with APS, the anti-*β*2GP1-IgG2 subclass had the highest contribution with 38%.

The finding of an elevated proportion of the IgG2 subclass in the patients with APS confirms former published data which also describe a skewing towards anti-*β*2GP1-IgG2 in APS [19, 23, 26, 27]. The authors detected an even higher contribution of anti-*β*2GP1-IgG2 (70.2% [26] and 87% [27], resp.). In contrast to our results, the IgG1 subclass was more prominent in the published studies described above. Importantly, in the cohort of the healthy infants IgG2 was relatively low. Therefore one may argue that anti-*β*2GP1-IgG2 is not just a marker of the disease but is also involved in the pathogenic action of the aPL autoantibodies. When we compared the anti-*β*2GP1-IgG subclass distribution between the three cohorts, we noticed a significant difference in the distribution concerning the children on one hand and NHD and patients with APS on the other. Healthy children showed a higher proportion of anti-*β*2GP1-IgG3 and a lower one of anti-*β*2GP1-IgG2. In NHD and patients with APS, it was the other way round. IgG1 and IgG4 did not significantly differ between healthy children and patients with APS or NHD. The predominance of anti-*β*2GP1-IgG3 in the healthy children is a hint to an immune response elicited by a proteinaceous antigen [26, 27, 34]. This supports the hypothesis that the harmless anti-*β*2GP1 in children are infection-induced with pathogen-derived proteins serving as trigger for their production. However, it is still elusive if the IgG3-dominated anti-*β*2GP1 response, found in the sera of healthy children, contributes to its low pathogenic potential. Alternatively, it can just be ascribed to the proceeding evolution of the infant's immune system typically associated with an elevated proportion of IgG3 antibodies [34, 35]. During childhood, the proportion of IgG3 continuously decreases to adult ranges usually reached at the age of 10–12 years. However, the above average elevated proportion of the anti-*β*2GP1-IgG3 (40%) in the sera of the healthy children may thus not just be due to the infant immune system. In particular when bearing in mind that normal adult levels of the IgG3 subclass are just around 6% [34, 36].

IgG3 is the IgG-subclass exerting the most effective activation of the complement cascade through the classical pathway [34]. Since complement activation is associated with the pathogenesis of APS [21, 32, 37], the high proportion of complement-activating anti-*β*2GP1-IgG3 in the children seems to contradict a possible protective effect against APS of these kinds of antibodies. However, the role of complement in the etiopathogenesis of autoimmune diseases is manifold as the complement components C1q, C2, and C4 contribute to the clearance of postapoptotic, secondary necrotic cells and their deficiency is, therefore, a risk factor for autoimmunity and autoinflammation [38]. In a similar manner, studies in mouse* in vivo* models of various autoimmune diseases demonstrated that IgG mediated tissue inflammation was blocked in mice deficient in activating Fc*γ*Rs, although the complement component C3 was still abundantly deposited in the tissue [39–41]. Thus, the contribution of complement deposits in tissue with respect to tissue inflammation remains to be established.

To obtain new insights into the involvement of the complement system in the pathogenesis of APS, we analysed the C1q- and C3c-binding to anti-*β*2GP1. We observed that anti-*β*2GP1 in the sera of the healthy children and in patients with APS similarly bound C1q, the first molecule of the classical pathway of complement activation [42]. Performing anti-*β*2GP1-IgM-ELISAs, we detected significantly higher IgM values in the patients with APS. One would expect a higher C1q-binding of the IgM positive sera, since this immunoglobulin binds and activates C1q more strongly than IgG. However, the children mainly harbour IgG3 autoantibodies, the most potent subclass for the activation of the classical complement pathway [34]. This may compensate for the lower IgM level in their sera and thus be responsible for the similar C1q-binding of both cohorts. This suggests that preconditions for complement activation via the classical pathway are comparable in both cohorts. We conclude that classical complement activation has a minor impact on the differential pathogenesis of aPL in healthy children and patients with APS.

What then is the culprit that induces their pathogenic potential?

Next we quantified the contribution of C3c to the immune complexes bound to *β*2GP1. C3c is a domain in the C3b molecule and one of its cleavage products created after cleavage by factor I of C3b [42, 43]. C3c not just represents a marker of complement activation but also serves as an opsonin, improving the clearance of bound targets. We observed significantly more C3c bound to anti-*β*2GP1 in the sera of the healthy children than in those of patients with APS. This reflects a better opsonization by C3c of the potentially harmful anti-*β*2GP1 immune complexes, anti-*β*2GP1-opsonized cells, or cellular fragments in the sera of the healthy children. Consequently, the anti-*β*2GP1 complexes can be cleared more efficiently by the powerful complement system of the healthy children. In patients with chronic inflammatory autoimmune diseases, complement is usually low, which may result from null alleles [44] or from complement consumption [38].

But why do these processes not cause inflammation and aberrant coagulation in the children? Apart from anti-*β*2GP1-IgG subclass distribution and distinct ability to activate the complement system, it is likely that other properties of the anti-*β*2GP1 in the sera of children contribute to their low pathogenicity. We hypothesized that an increased sialylation of the anti-*β*2GP1-IgG in healthy individuals renders them harmless for their hosts. IgG is usually N-glycosylated at the CH2-domain of its Fc-part. This N-glycan crucially modifies the effector functions the IgG molecule is endowed with [45–47]. This biantennary, core-fucosylated oligosaccharide carries a variable amount of sugar monomers attached to both arms [28, 48]. IgG antibodies terminating with sialic acids on one or both antennae have been shown to behave anti-inflammatorily after infusion of IVIG [29, 31]. The signal exerted by the terminal sialic acid is dominant since the fraction of sialylated IgG in healthy plasma is just 4 to 14% [28, 46, 49].

In mice, these effects are mainly ascribed to decreased binding of sialylated IgG to activating Fc*γ*-receptors and an upregulation of the inhibitory Fc*γ*RIIB [29, 31]. We observed that anti-*β*2GP1-IgG isolated from sera of healthy children was significantly more sialylated than that from symptomatic patients with APS. One may predict that these autoantibodies, in complex with *β*2GP1, show low affinity for activating Fc*γ*Rs and, therefore, do not provoke proinflammatory and procoagulant effector functions. Fc*γ*RIIA is an example for an activating receptor. It is located on a plethora of cells. On the surfaces of platelets, it mediates activation by immune complexes. The activation is accompanied by platelet aggregation and the release of mediators such as the prothrombotic Thromboxane A2 [50]. Consecutively, this cascade can result in thrombus formation [51, 52]. Sialylated anti-*β*2GP1-IgG is unable to bind to and to activate platelets via Fc*γ*RIIA.

Further mechanisms involved in thrombus formation in APS comprise the interaction of anti-*β*2GP1 with various pattern recognition receptors [53] and its interference with regulatory functions of molecules involved in the coagulation process (protein C, prothrombin, or tissue factor [52, 54]). Direct binding to *β*2GP1 on endothelial cells (EC) shifts their phenotype towards coagulation and inflammation [54]. Considering that Fc*γ*R-binding is involved in many of these mechanisms, it seems likely that a high sialylation of anti-*β*2GP1-IgG leads to a low pathogenicity of these autoantibodies and does not cause APS. Therefore, we conclude that the higher sialylation of anti-*β*2GP1-IgG in the sera of the healthy children plays a major role in protecting them from typical complications of APS. This hypothesis is further supported by the observation that antiphospholipid antibodies from sera of asymptomatic adult carriers are more sialylated than those from patients with APS. A high sialylation status of antiphospholipid antibodies seems to prevent the development of symptoms of APS also in these individuals.

We conclude that a plethora of factors contributes to the low pathogenicity of anti-*β*2GP1-IgG in the sera of healthy children and asymptomatic adults. We observed a distinct spectrum of IgG-subclasses compared to patients with APS and a better binding of C3c indicating a more efficient clearance of potentially harmful anti-*β*2GP1. The hypersialylation of the anti-*β*2GP1-IgG is prone to ameliorate their inflammatory and procoagulatory effects in healthy carriers, whereas hyposialylation and inflammation mutually precipitate the antiphospholipid syndrome.

## Supplementary Material

Supplementary Figure 1. The diagram describes the selection process of those sera investigated regarding their IgG-subclasses. First, anti-*β*2GP1-IgG titers were measured in all sera. Then we defined the cut-off as the 97.5%-quantile of NHD cohort. The IgG-subclasses were measured in those sera exceeding this threshold (12 PAPS, 6 SAPS and 13 Children). To define the number of seropositive samples of the respective IgG-subclasses, we proceeded as follows: we calculated the mean OD of the corresponding IgG-subclass in the NHD cohort and added two times its standard deviation (SD) (mOD[NHD] + 2 x SD[NHD]). Sera exceeding this value were then defined seropositive for this specific IgG-subclass. The results are shown in Table 4. mOD = (milli)optical density; NHD = normal healthy donor; PAPS = primary antiphospholipid syndrome; SAPS = antiphospholipid syndrome with systemic lupus erythematosus; SD = standard deviation.

## Figures and Tables

**Table 1:** Antibodies employed for each ELISA.

ELISA	Detection-antibody	Dilution
*Anti-β2GP1-IgG *	HRP-conjugated rabbit F(ab′) 2 anti-human-IgG^*∗*^	1 : 20.000
*Anti-β2GP1-IgG subclasses *	HRP-conjugated mouse anti-human IgG1, 2, 3 and 4 (Clones HP6001, HP6002, HP6050 & HP6025)^*∗*^	1 : 20.000
*Anti-β2GP1-IgM *	HRP-conjugated goat anti-human-IgM^*∗*^	1 : 20.000
*Anti-β2GP1-bound C3c *	HRP-conjugated sheep anti-C3c^*∗∗*^	1 : 500
*Anti-β2GP1-bound C1q *	HRP-conjugated sheep anti-C1q^*∗∗*^	1 : 500

*∗*: purchased from Southern Biotech, Birmingham, Alabama, USA.

*∗∗*: purchased from Abcam, Cambridge, United Kingdom.

HRP = horseradish peroxidase.

**Table 2:** Detection reagents for lectin ELISA.

SNA-ELISA	
Primary detection reagent	Biotinylated SNA-lectin (Galab Technologies, Geesthacht, Germany)
Secondary detection reagent	HRP-conjugated streptavidin (Jackson ImmunoResearch laboratories, Newmarket, Suffolk, United Kingdom)
Dilution	1 : 10.000

SNA = *Sambucus nigra* agglutinin; HRP = horseradish peroxidase.

**(a):** 

	IgG1 [mOD]	IgG2 [mOD]	IgG3 [mOD]	IgG4 [mOD]
NHD	24	64	27	67
Children	69	77	212	210
PAPS	30	85	45	72
SAPS	28	74	55	173
APS	29	81	48	106

**(b):** 

	IgG1	IgG2	IgG3	IgG4
Children versus NHD	<0.0001	n.s.	<0.0001	n.s.
Children versus PAPS	<0.0001.	n.s.	<0.001	n.s.
Children versus SAPS	<0.001	n.s.	<0.001	n.s.
APS versus NHD	n.s.	n.s.	n.s.	n.s.
PAPS versus SAPS	n.s.	n.s.	n.s.	n.s.

OD = optical density 450 nm; PAPS = primary antiphospholipid syndrome; SAPS = secondary antiphospholipid syndrome; APS = PAPS and SAPS; NHD = normal healthy donors; n.s. = not significant.

**Table 4:** Number (frequency) of seropositive samples of the anti-*β*2GP1-IgG subclasses.

Number of seropositive samples (>mOD_[NHD] _+ 2 × SD_[NHD]_)				
	IgG1 (>2,9 mOD)	IgG2 (>7,9 mOD)	IgG3 (>8,5 mOD)	IgG4 (>31,9 mOD)
NHD (*n* = 17)	0 (0%)	0 (0%)	1 (6%)	1 (6%)
Children (*n* = 13)	13 (100%)	2 (15%)	8 (62%)	2 (15%)
PAPS (*n* = 12)	6 (50%)	5 (42%)	0 (0%)	1 (8%)
SAPS (*n* = 6)	1 (17%)	2 (33%)	0 (0%)	1 (17%)
APS (*n* = 18)	7 (39%)	7 (39%)	0 (0%)	2 (11%)

OD = optical density 450 nm; PAPS = primary antiphospholipid syndrome; SAPS = secondary antiphospholipid syndrome; APS = PAPS and SAPS; NHD = normal healthy donors. The cut-off for seropositivity was calculated from the mean OD of the corresponding anti-*β*2GP1-IgG subclass of the NHD cohort and its standard deviation (SD) according to the formula: mean OD_[NHD] _+ 2 × SD_[NHD]_. The respective cut-off OD values are indicated in parentheses below the anti-*β*2GP1-IgG subclasses.

**Table 5:** Cohorts studied with the lectin ELISA.

Cohort	Abbreviation	Number of samples
Patients with primary APS	PAPS	12
Patients with APS and SLE as underlying disease	SAPS	10
Patients with SLE without symptoms of APS harbouring circulating aPL	SLE + aPL	15
Asymptomatic carriers of aPL	aaPL	6
Healthy children with circulating anti-*β*2GP1	—	16

APS = antiphospholipid syndrome; aPL = antiphospholipid antibodies; SLE = systemic lupus erythematosus.
