# Supplementary figures and images for: The Pathogenicity of Anti-β2GP1-IgG Autoantibodies Depends on Fc Glycosylation
Source: J Immunol Res. 2015 Jun 22;2015:638129. doi: 10.1155/2015/638129 (PMC4491572; doi:10.1155/2015/638129)

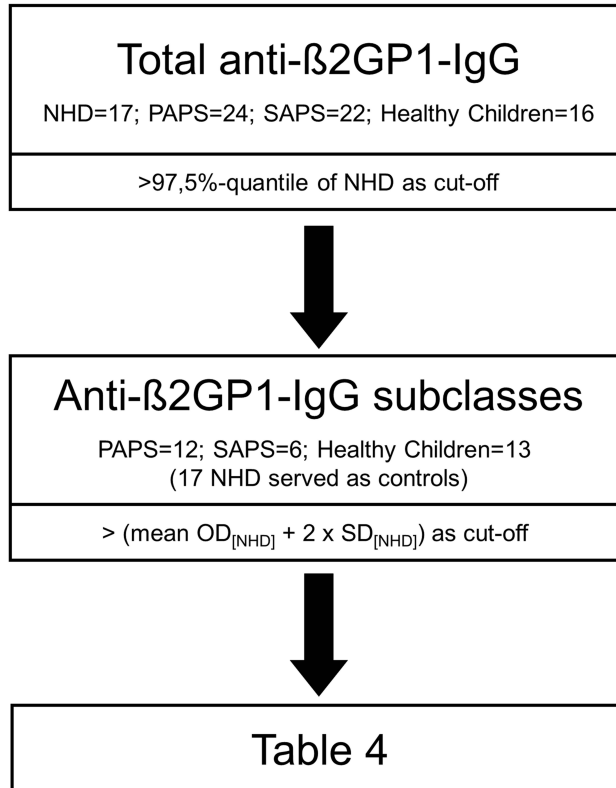

Supplement: Supplementary file 1 — Supplementary Figure 1. The diagram describes the selection process of those sera investigated regarding their IgG-subclasses. First, anti-β2GP1-IgG titers were measured in all sera. Then we defined the cut-off as the 97.5%-quantile of NHD cohort. The IgG-subclasses were measured in those sera exceeding this threshold (12 PAPS, 6 SAPS and 13 Children). To define the number of seropositive samples of the respective IgG-subclasses, we proceeded as follows: we calculated the mean OD of the corresponding IgG-subclass in the NHD cohort and added two times its standard deviation (SD) (mOD[NHD] + 2 x SD[NHD]). Sera exceeding this value were then defined seropositive for this specific IgG-subclass. The results are shown in Table 4. mOD = (milli)optical density; NHD = normal healthy donor; PAPS = primary antiphospholipid syndrome; SAPS = antiphospholipid syndrome with systemic lupus erythematosus; SD = standard deviation. [file 638129.f1.pdf]
